# Association Between Fear of COVID-19 and Emotional Distress in Nurses With Mediating Role of Socio-Demographic Features

**DOI:** 10.3389/fpsyg.2021.734623

**Published:** 2021-10-01

**Authors:** Mueen Abid, Maryam Riaz, Zaqia Bano, Tahira Parveen, Muhammad Umar Fayyaz, Halima Sadia Qureshi

**Affiliations:** ^1^Department of Psychology, University of Gujrat, Gujrat, Pakistan; ^2^Department of Psychology, National University of Medical Sciences Rawalpindi, Rawalpindi, Pakistan

**Keywords:** COVID-19, emotional distress, linear aggression, structural equation modeling, depression, anxiety, stress, socio-demographic features

## Abstract

**Objectives:** To determine the predictive association between fear of COVID-19 and emotional distress (depression, anxiety, and stress) in frontline and non-frontline nurses. To explore the mediating role of socio-demographic features.

**Methods:** Correlational cross-sectional research design was implied. A total of 500 on-duty male and female, frontline and non-frontline, nurses were included from five major hospitals in Gujrat (Aziz Bhatti Shaheed Hospital, City Hospital, Doctors Hospital, Akram Hospital, and Gujrat Hospital). Fear of COVID-19 scale and the Urdu version of depression, anxiety, and stress scale - 21 (DASS-21) were used to measure variables of interest. Descriptive statistics, structural equation modeling (SEM), linear regression, and *t*-test were carried out using Statistical Package for Social Sciences (SPSS) 21.

**Result:** Structural equation modeling (SEM) revealed a significant predictive link between fear of COVID-19 and depression, anxiety, and stress (goodness of model fit; NFI = 0.93, GFI = 0.914, AGFI = 0.93, CFI = 0.936, and IFI = 0.936). Furthermore, a significant mediating effect of certain demographic features was discovered by SEM (CMIN/DF = 1.11, NFI = 0.94, TLI = 0.98, GFI = 0.08, AGFI = 0.93, RMSEA = 0.029, CFI = 0.99, and IFI = 0.99). Results of linear regression analysis also revealed a momentous predictive association between fear of COVID-19 and emotional distress (*R* = 0.860). In comparative analysis, the results of t-test explored the statistical significant difference in fear of COVID-19 and emotional distress between frontline (mean = 25.775, 36.147 and SD = 1.75, 2.23) and non-frontline nurses (mean = 21.702, 27.353 and SD = 4.607, 10.212), with *t*_(130)_ =7.111, 6.92.

**Conclusion:** Managing the mediating effect of demographic characteristics and reducing the fear of COVID-19 can help nurses to overcome emotional distress, such as depression, anxiety, and stress. Further, this will increase the productivity among nurses.

## Introduction

Coronavirus disease (COVID-19) is an illness which unsympathetically affected public health and overall circumstance across the border. In November 2019, a sickness resembling pneumonia appeared in Wuhan, China, which was later named by the World Health Organization (WHO) as Coronavirus Disease 2019 or COVID-19. Fear of COVID-19 has been interpreted as a perceived threat to the disease, which has produced significant financial destruction and crushed the community mental health in several nations (WHO, 2020).

According to the National Institute of Health Islamabad (2020), in Pakistan, the Ministry of Health, the government of Pakistan in Karachi, and the Pakistan Federal Ministry of Health in Islamabad first reported two cases of COVID-19 on February 26, 2020. At present, the number of cases has escalated and created the worst situation. Among the provinces of Pakistan, the highest number of cases was reported in Punjab and Sindh (Waris et al., 2020). Gujrat district reported the highest number of confirmed cases among all the districts in Punjab. The first case of COVID-19 was reported on March 16, 2020, and a total of 104 cases were reported in a single day on July 9, 2020 (Imtiaz et al., 2020).

It is useful to discuss the services of nurses and other medical personnel during the pandemic because around the world they have been playing an important part in the times of pandemic and crisis. In Pakistan, 63 healthcare workers lost their lives fighting the COVID-19 pandemic (Tribune, 2020). International Council of Nurses (ICN) has highlighted the services that nurses provide under life-threatening conditions during a crisis and in adverse circumstances. The extraordinary burden created by the epidemic on every nation's healthcare structure has presented numerous trials to the nurses and other medical workers, which may cause disturbances to their mental health and overall work performance by creating depressive, anxious, and stressful situations (Mo et al., 2020). So, it is vital to preserve the emotional and mental fitness of the healthcare staff and nurses (Catton, 2020; Mo et al., 2020). Studies investigating mental health problems and the associated factors among the frontline nurses during an outbreak are very essential to plan the strategies needed to combat emotional distress. Subsequently, studies exploring the nursing labor force during the COVID-19 pandemic are of paramount importance (Thapa et al., 2020). This is because nurses who have worked with COVID-19 patients are more vulnerable to experience emotional distress that is a negative emotional reaction such as depression, anxiety, and stress (Kameg, 2020). Another mental health study involving 1146 medical staff from Indonesia, India, Singapore, Malaysia, and Vietnam found a high prevalence of stress disorders with a low diagnosis of depression and anxiety. Non-medically trained workers experienced a higher level of mental health concerns compared to medically trained workers (Chew et al., 2020a).

A survey conducted on 906 medical staff revealed a momentous relation between COVID-19 and mental health outcomes in healthcare staff. Further, it was reported that 5.3% of the participants exhibited moderate to very severe levels of depression, 8.7% of the participants showed moderate to profound levels of anxiety, 2.2% of the participants showed moderate to extremely severe levels of stress, and 3.8% of the participants exhibited moderate to severe levels of emotional distress (Chew et al., 2020b). Additionally, a greater occurrence of emotional distress, such as anxiety and depression, was observed among nurses who worked with COVID-19 patients when compared to other medical workforces (Pappa et al., 2020).

Another study in Italy measured the levels of stress, depression, and anxiety in healthcare workers during the COVID-19 outbreak. The study concluded that the protective measures against the effects of mental health in the general population are well-known, but there is still a lack of research on the outcomes with regard to the medical staff. Furthermore, the study found that ~10% of the frontline medical workers reported moderate to very severe indications of depression, anxiety, and stress during the COVID-19 outbreak (Lenzo et al., 2021).

A study conducted among Pakistani medical workers has found that the fear of COVID-19 causes a high level of anxiety among medical personnel and patients with hepatitis. This study shows that the promotion of stable mental health plays an important role in boosting the immune system, and further demands the need for positive steps to be taken to reduce such epidemics and anxiety among medical workers (Rafique et al., 2021).

Socio-demographic features are among the strongest determinants of people's attitude toward their physical and mental health (Parra, 1985). Findings have revealed that during the COVID-19 outbreak, demographic characteristics such as age, education, socio-economic status, and nature of job are highly significant to account for the negative effects on psychological health (Holmes et al., 2020).

All over the world, various studies have explored the effect of COVID-19, but unfortunately only a few researches have revealed the role of demographics in emotional problems among medical workers, arising due to the fear of COVID-19 (Thapa et al., 2020). To bridge this gap, the current study aims to explore a predictive association between the fear of COVID-19 and emotional distress among frontline and non-frontline nurses with the mediating role of demographic characteristics. This study therefore looks at the health professionals, counselors, authorities involved in nursing training, policy makers, and senior medical workers who are concerned about protecting and enhancing the emotional and mental health of nurses by reducing the fear of COVID-19 and other mediating factors.

**Figure 1:**
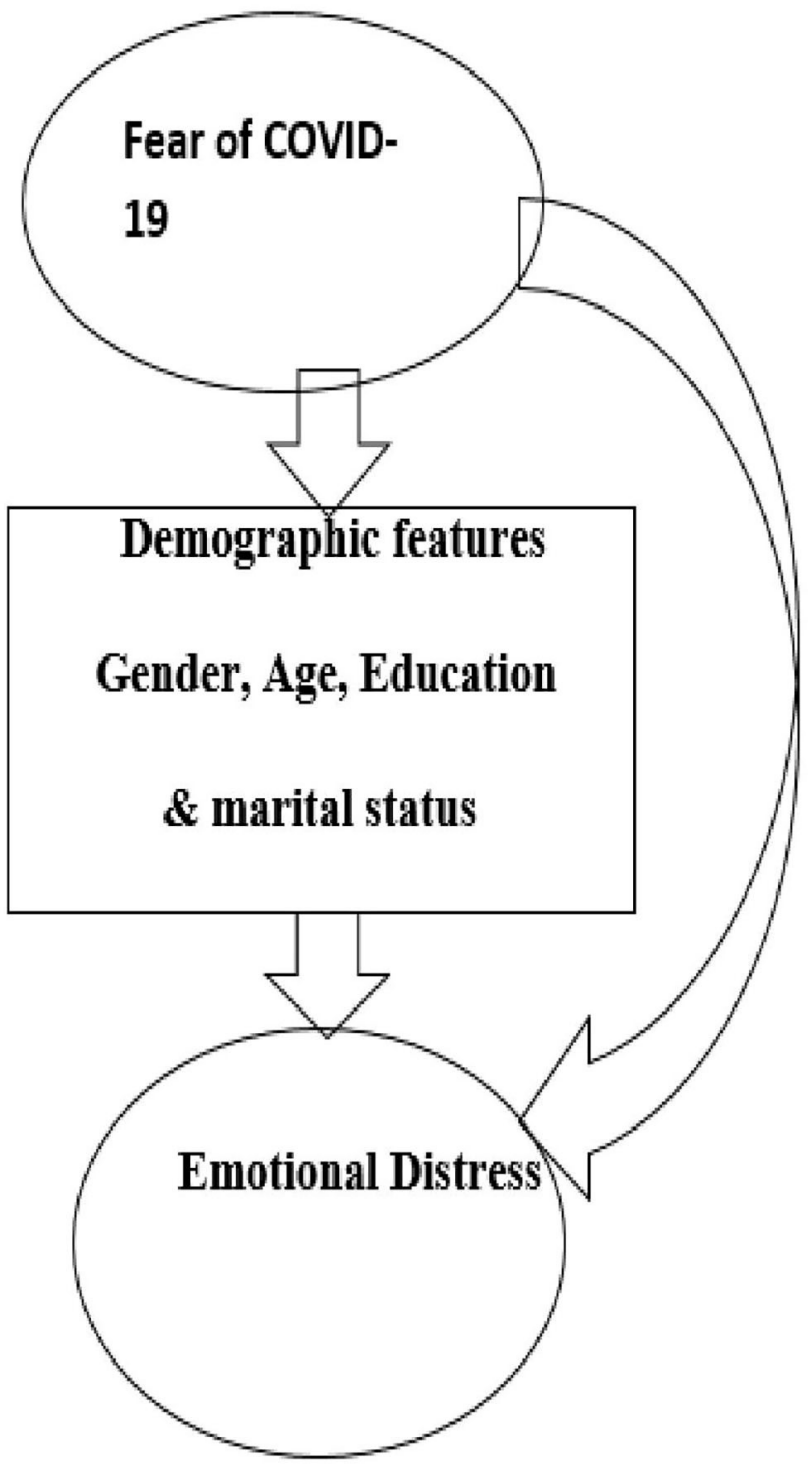
Representation of proposed model of this study.

The proposed model of this study is presented in Figure 1.

## Method

### Research Design

This is a cross-sectional study, which involves exploring and accumulating data about a study populace at one point in time. Cross-sectional design has been labeled as portraits of the inhabitants about which data is collected (Lavrakas, 2012).

### Population, Sample, and Location of Study

This study was conducted in Gujrat, Pakistan during the period from February 15 to October 13, 2020. The population of study included all the men and women employees working as full-time (regular employees) and part-time (contractual employees) nursing staff in public and private sector hospitals in Gujrat, Pakistan during the COVID-19 pandemic. The sample of study comprised 500 nurses, including 200 frontline and 300 non-frontline nurses, in Gujrat, Pakistan. Data was collected from all the available full-time and daily wage nursing staff, both men and women, from five major hospitals in Gujrat (Aziz Bhatti Shaheed Hospital, City Hospital, Doctors Hospital, Akram Hospital, and Gujrat Hospital). As only these five hospitals provided services during the first spike of COVID-19, all the nursing staff from these hospitals were included in the present study.

### Instrument of Study

Instrument of study comprises two parts. In the first part, a self-constructed demographic information sheet was used for inquiring information regarding gender, age, education, marital status, duty timings, job status, and attending to COVID-19 patients.

In the second part, fear of COVID-19 scale and depression, anxiety, and stress scale - 21(DASS-21) were administered to measure the variables of interest. Fear of COVID-19 scale was used to measure the fear of COVID-19 in nurses (Ahrsu et al., 2020). It is a 7-item scale measuring two dimensions of fear. Items 1, 2, 4, and 5 gauge emotional fear reaction, while items 3, 6, and 7 measure symptomatic reaction of fear. Score range is 7–35; a high score depicts a high level of fear, while a low score depicts a low level of fear regarding COVID-19. In the current study, reliability for the fear of COVID-19 scale is 0.75. On the other hand, Urdu version of DASS-21 translated by Aslam (2007) was used to measure emotional distress. DASS-21, which was used in the current study, is a brief and more comprehensive form of DASS-42 (Aslam, 2007). DASS-21, which is a 21-items scale, measures three dimensions of emotional distress (depression, anxiety, and stress). Each sub-scale contains seven items. In the current study, a high internal consistency of the scale with a Cronbach's alpha of 0.85 was reported. The use of DASS-21 was reported by different studies during the COVID-19 outbreak in Asia. A longitudinal survey was conducted to study the psychological health of 1738 participants from 190 cities in China during the COVID-19 outbreak. Mental health of the participants was measured using the DASS-21. It was clarified that DASS-21 is grounded on a multi-dimensional model of pathology with a number of features including a holistic distress paradigm (Wang et al., 2020a). Similarly, a cross-section study in Philippines discovered the psychological effects of COVID-19 using DASS-21, which is a brief and comprehensive measure to gauge psychological and emotional distress (Tee et al., 2020).

### Procedure

Procedure implemented in this study was observed and sanctioned by the Advanced Studies and Research Board (ASRB), University of Gujrat, Pakistan. All commendations of the panel were assimilated in the current investigation. The setting of current study included five big public and private hospitals in the district of Gujrat, Pakistan (Aziz Bhatti Shaheed Hospital, City Hospital, Doctors Hospital, Akram Hospital, and Gujrat Hospital). Prior to approaching the participants of study, consent was taken from the concerned authorities of the respective hospitals from where the samples were to be drawn, and a list of names and contact numbers of the nursing staff was also taken from those hospitals. After obtaining an online informed consent from the participants, the researchers electronically collected data on above said instruments according to the availability of participants. This study approached 500 participants, of which 65 participants declined to participate due to their unwillingness. To ensure the accuracy of data, the participants were educated about the aim of research, its implication, and voluntary basis of the study.

### Ethical Consideration

This study was conducted in accordance with the ethical guidelines of Human Research Committee, University of Gujrat, Pakistan. The participants were briefed about the purpose of study and the complete procedure, and it was made clear that their participation in the study was completely voluntary. Prior to data collection, a written informed consent was obtained from each participant. Further, the confidentiality and privacy of data were ensured to the participants.

### Statistical Analysis

Statistical analysis was performed by Statistical Package for Social Sciences (SPSS, 23Version) and Analysis of Moment Structure (AMOS 21 Version). Descriptive statistics was used to describe data in the form of frequencies, percentage, and graphical representation. In inferential statistics, SEM was used to check the overall model fitness of data and predictive association between fear of COVID-19 and emotional distress in nurses. Further, simple linear regression analysis was performed to check the portion of variation in emotional distress explained by fear of COVID-19, whereas in comparative analysis, independent sample *t*-test was applied to investigate statistical significant difference between frontline and non-frontline nurses with regard to fear of COVID-19 and emotional distress.

## Results

Frequencies and the percentage of demographic characteristics of the participants were computed. Majority of the participants were women (71%) compared to men (29%), and the age category with the highest score was 36–45 years, as 250 participants (50%) belonged to this age group. Among the remaining, 210 participants (42%) belonged to the age range of 25–35 years, while 40 participants (8%) belonged to the age range of 46–55 years. In terms of educational qualification, 38.4% of the participants had completed inter-level education, while only 2.4% (12 participants) were highly qualified. The number of participants with full-time employment were 330 (66%), while the remaining 170 participants (34%) were employed in part-time jobs. It was also revealed that 200 participants (40%) had worked with COVID-19 patients and 300 participants (60%) had not attended to such patients (Table 1).

**Table 1:** Demographic profile of participants (*N* = 500).

**Variables**		**Categories frequencies**	**Percentage**
Gender	Male	145	29
	Female	355	71
Age	25–35	210	42
	36–45	250	50
	46–55	40	8
Education	Matriculation	108	21.6
	Intermediate	192	38.4
	Bachler	188	37.6
	Master	12	2.4
Nature of job	Full time	330	66
	Part Time	170	34
Attended COVID-19 patient	Yes	200	40
	No	300	60

Means and standard deviations (SD) of the variables of the study were computed. The mean score for the fear of COVID-19 scale was 23.92 and SD was 3.92, which was above the average score. For the emotional distress (DASS-21) scale, the composite score was 32.15 and SD was 8.30 (Table 2).

**Table 2:** Descriptive statistics of the variables of the study (*N* = 500).

**Variables**	***N***	**Minimum**	**Maximum**	**Mean**	***SD***
Fear of COVID-19	500	11.29	28.71	23.92	3.92
Emotional- Distress (DASS)	500	7.00	38.10	32.15	8.30

To identify internal consistency of the data in the current study, reliability analysis was performed to find Cronbach's alpha. Based on the analysis, reliability score for fear of COVID-19 scale was 0.75, while the scale of emotional distress (DASS) showed a Cronbach's alpha value of 0.85. So, the reliability analysis of both measurements (0.75 and 0.85) revealed high internal consistency of scales in the current study (Table 3).

**Table 3:** Cronbach's alpha scores for each measurement.

**Measurement**	**No of items**	**Cronbach's alpha scores**
Fear Of COVID-19	07	0.75
DASS	21	0.85

**Figure 2:**
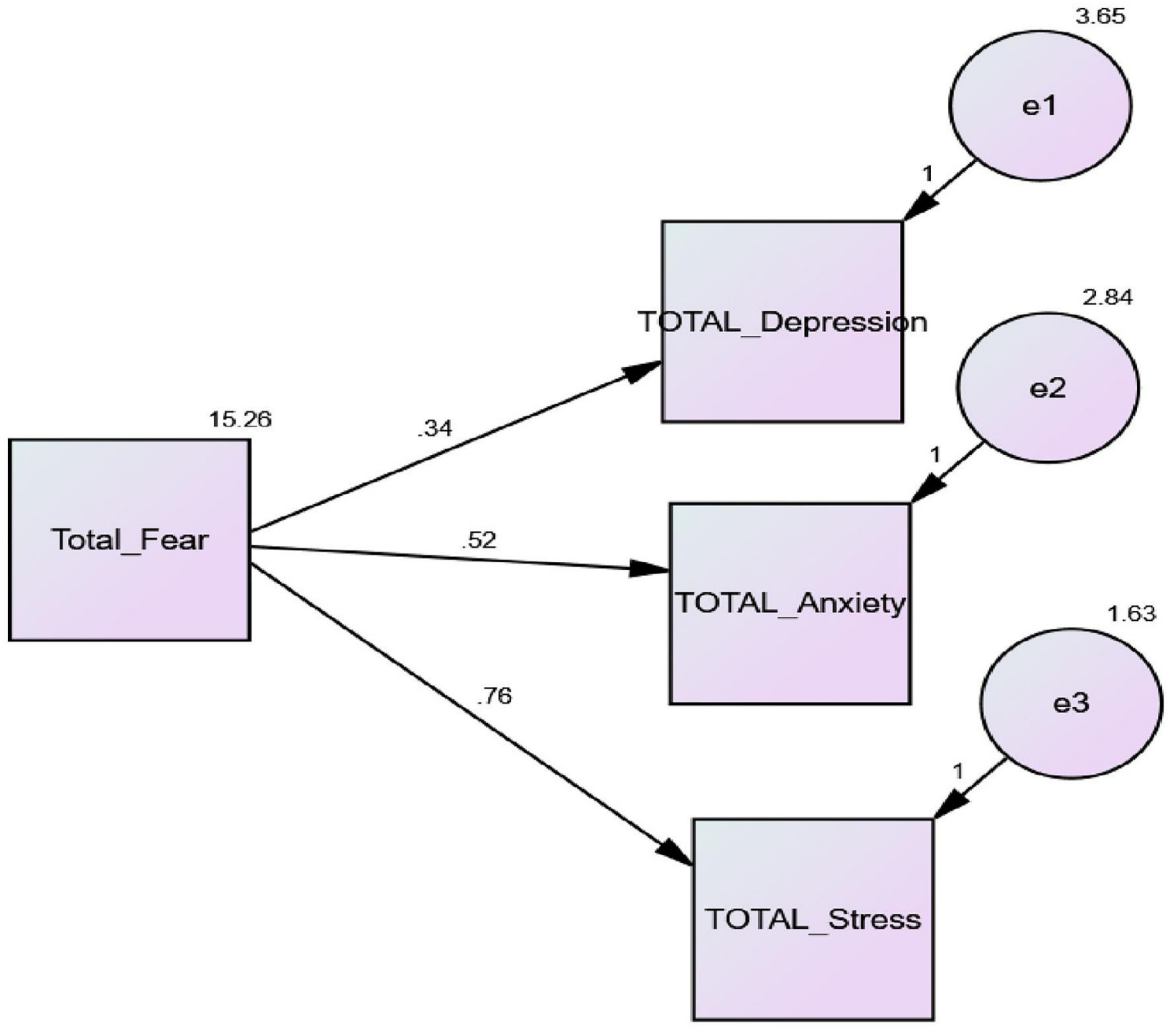
SEM model of predictive association between fear of COVID-19 and sub-scales of depression, anxiety, and stress (DASS).

This study identified that fear of COVID-19 was a significant predictor of stress, depression, and anxiety in nurses. Furthermore, regression weights indicated that fear of COVID-19 accounted for 34% variance in depression, 62% in anxiety, and 70% in stress, respectively (Figure 2).

SEM was implied to check the predictive association between fear of COVID-19 and sub-scales of DASS (depression, anxiety, and stress). The result showed the goodness of model fit as the values of NFI = 0.93, GFI = 0.914, AGFI = 0.93, CFI = 0.936, and IFI = 0.936 were highly acceptable. The results of the SEM model showed a significant predictive association between fear of COVID-19 and depression, anxiety, and stress. Furthermore, the model of study is accepted (Table 4).

**Table 4:** Model fit summary of structural equation modeling (*N* = 500).

**NFI**	**GFI**	**AGFI**	**CFI**	**IFI**
0.93	0.914	0.93	0.936	0.936

**Figure 3:**
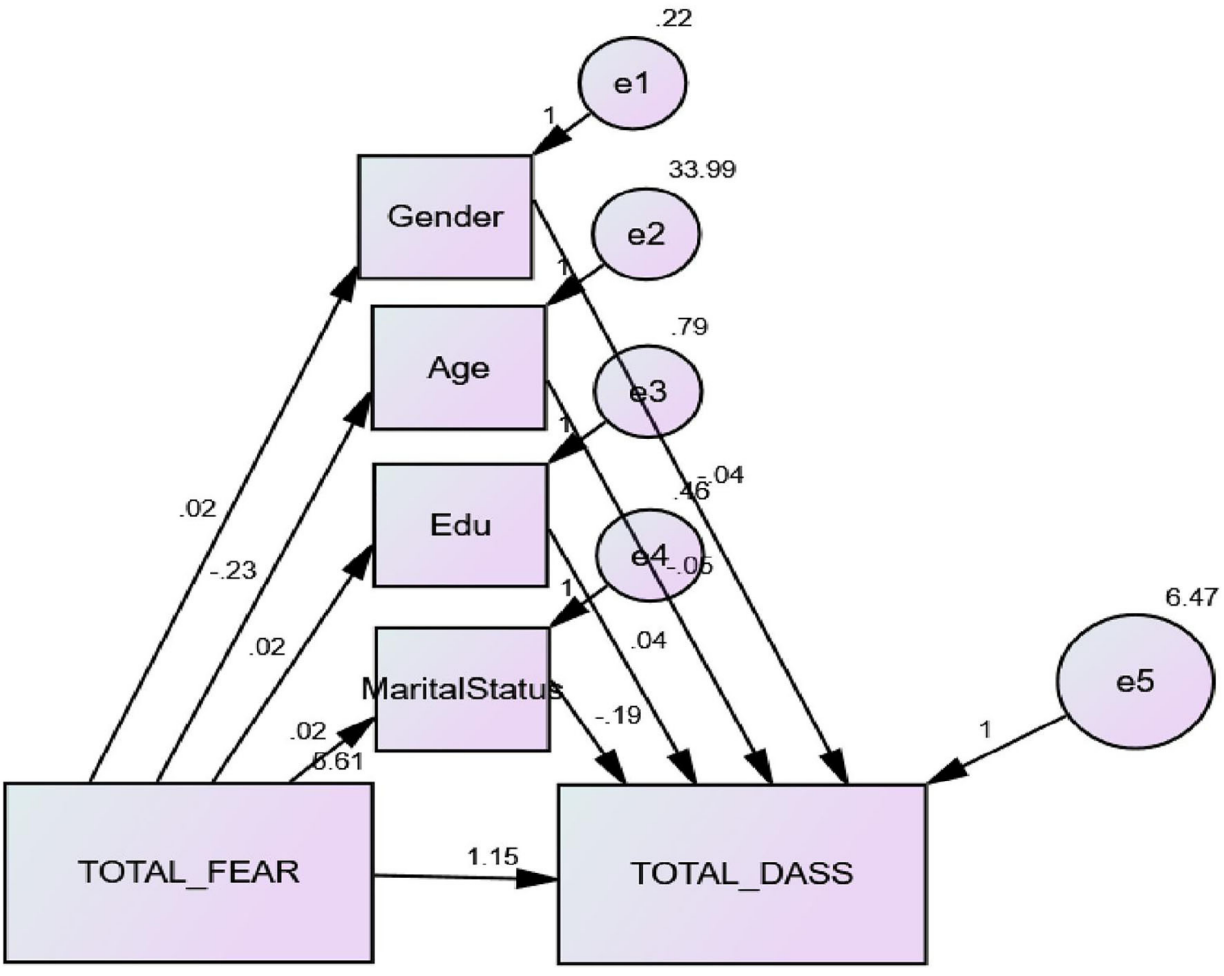
Mediation model of predictive association between study variables (*N* = 500).

Findings of this study specified a significant mediating role of demographic characteristics, such as gender, age, education, and marital status, in the predictive association between fear of COVID-19 and emotional distress in nurses (Figure 3).

SEM was implied to check the predictive association between fear of COVID-19 and emotional distress and the mediating role of socio-demographic variables. The result displayed goodness of fit indicators for the planned SEM model as the values CMIN/DF = 1.11, NFI = 0.94, TLI = 0.98, GFI = 0.08, AGFI = 0.93, RMSEA = 0.029, CFI = 0.99, and IFI = 0.99 were highly acceptable. Further, the results of the SEM model showed a strong predictive association between fear of COVID-19 and emotional distress and the link was mediated by socio-demographic variables such as gender, age, education, and marital status. Hence, the model of study is accepted (Table 5).

**Table 5:** Model fit summary of structural equation modeling (*N* = 500).

**CMIN/DF**	**NFI**	**TLI**	**GFI**	**AGFI**	**RMSEA**	**CFI**	**IFI**
1.11	0.94	0.98	0.98	0.93	0.029	0.99	0.99

A simple linear regression was carried out to predict emotional distress based on fear of COVID-19. Fear of COVID-19 is an independent variable, whereas emotional distress is a dependent variable. A significant regression equation was found *F*_(1, 130)_ = 797.027, *p* < 0.001 with an *R*^2^ value of 0.860. The results specified that the model explained 86% of the variance and that the model was significant. Furthermore, participants predicted emotional distress is equal to −14.865 + 1.964 (fear of COVID-19) and emotional distress was measured in the terms of depression, anxiety, and stress. Participant's emotional distress increased by 1.964 when fear of COVID-19 increased. So, it was found that fear of COVID-19 significantly predicted emotional distress (β1 = 1.97, *p* < 0.001).

The final predictive model was: −14.85 + (−1.95^*^ fear of COVID-19) (Table 6).

**Table 6:** Fear of COVID-19 as the predictor of emotional distress among nurses (*N* = 500).

**Variables**	**β**	**SE**	**β**	***R***	***R*** **-square**	***p*** **-value**
Constant	−14.846	1.687				
Fear of COVID-19	1.964	0.070	0.927	0.927	0.860	0.000

*p < 0.001*.

Independent sample t-test was conducted to compare the fear of COVID-19 and emotional distress between nurses who worked and those who did not work with COVID-19 patients. The result showed a statistically significant difference at the 0.001 level of significance, between the nurses who worked (mean = 25.775, 36.147 and SD = 1.75, 2.23) and those who did not work with COVID-19 patients (mean = 21.702, 27.353 and SD = 4.607, 10.212), with *t*_(498)_ = 7.111, 6.92, respectively. Overall results revealed that the nurses who worked with COVID-19 patients had a high level of fear of COVID-19 and emotional distress (mean = 25.775, 36.147 and SD = 1.75, 2.23) compared to nurses who did not work with COVID-19 patients (mean = 21.702, 27.353 and SD = 4.607, 10.212) (Table 7).

**Table 7:** Comparison of fear of COVID-19 and emotional distress between nurses who worked and did not work with COVID-19 patients (*N* = 500).

**Outcome**	**Group**									
	**Attended COVID-19 patient**			**Not attended COVID-19 patient**						
	***M***	***SD***	***N***	***M***	***SD***	***N***	**95% CI for mean difference**	***t***	**Df**	***p***
Fear of COVID-19	25.775	1.75	200	21.702	4.60	200	6.3476, 11.241	7.11	498	.000
DASS	36.147	2.23	300	27.353	10.2	300	2.910, 5.236	6.92	498	.000

*p < 0.001*.

Multiple regression analysis was implied to check the predictive effect of gender on fear of COVID-19 and emotional distress. The result depicted that there was no predictive effect of gender on fear of COVID-19 and emotional distress (Table 8).

**Table 8:** Summary of multiple regression analysis between gender, fear of COVID-19, and emotional distress (*N* = 500).

**Variables**	**β**	**SE**	**β**	***R***	***R*** **-square**	***p*** **-value**
Constant (Gender)	−0.874	1.529		−0.050		0.569
Fear of Covid-19	1.963	0.070		0.927		0.463
*R*	0.050					
*R* ^2^	0.003					
ΔR	−0.005					
*F*	0.326					

To identify the predictive association between fear of COVID-19 and emotional distress (depression, anxiety, and stress), linear regression was implied. The results showed a significant predictive association between fear of COVID-19 and emotional distress (stress, depression, and anxiety) in nurses. Moreover, the above statistics depicted that fear of COVID-19 explained 55% variation in depression, 66% variation in anxiety, and 49% variation in stress (Table 9).

**Table 9:** Fear of COVID-19 as the predictor of emotional distress (depression, anxiety, and stress) in nurses (*N* = 500).

**Variables**	**β**	**SE**	**β**	***R***	**R-square**	***p*** **-value**
Depression	1.766	0.687	0.721	0.721	0.553	0.000
Anxiety	1.675	0.711	0.827	0.581	0.660	0.000
Stress	1.544	0.523	0.827	0.581	0.494	0.000

*p < 0.001*.

## Discussion

This study was conducted during the peak of COVID-19 pandemic in Pakistan, and hence the outcomes of the current study provide the first insight about the predictive role of fear of COVID-19 on depression, anxiety, and stress in nurses. Nurses were the most vulnerable group during that time and there was a dire need to explore the fear of COVID-19 and emotional distress. A study documented that frontline medical workers were at highest risk to experience psychological problems because they directly treated COVID-19 patients and frequently observed distressed and deceased patients, which impacted their psychological well-being (Ahrsu et al., 2020). A similar study was conducted on emotional distress and mental health issues in medical workers of Singapore during the COVID-19 outbreak. Findings of this study highlighted that mental health problems were mostly faced by untrained non-medical workers when compared to trained medical workers. This was because training and counseling enhanced the coping abilities and stress management skills in medical workers, which further helped to reduce the risk of various mental health problems (Satici et al., 2020; Tan et al., 2020).

In the present study, emotional distress was measured in terms of depression, anxiety, and stress. The results of SEM revealed that the fear of COVID-19 played a key role in determining depression, anxiety, and stress in nurses. This is because fear of COVID-19 is one of the major causes of emotional distress. Furthermore, the results of linear regression analysis also depicted that 86% of the change in emotional distress was attributed to the fear of COVID-19 in nurses. Among the limited studies available on the population of nurses, this association was also proved by prior studies comprising the general public. For example, a cross-sectional study of 175 nurses in Lahore, Pakistan, measured the levels of stress, depression, and anxiety using DASS-21. This study showed that high levels of fear of COVID-19 were powerfully associated with negative emotional responses such as anxiety, depression, and stress. Furthermore, this study suggested that there was an urgent need for effective mental health training and counseling programs to promote mental health and diminish the chronic effects of COVID-19 pandemic (Riaz et al., 2021). A recent study revealed that a fear of COVID-19 had a substantial progressive association with emotional distress (anxiety, depression, and stress; Bakioglu et al., 2020).

Current study added new knowledge about some demographic features which served as a mediating factor in the predictive association between fear of COVID-19 and emotional distress. Some important demographics such as age, education, and marital status play a significant mediating role in enhancing the above said predictive association. However, this study represented no predictive effect of gender on fear of COVID-19 and emotional distress. Thus, by controlling and managing the relevant demographic features, fear of COVID-19 can be reduced which would be helpful to lessen the emotional distress in nurses. Prior studies confirmed the finding of the current study; for instance, a cross-sectional study comprising 600 participants in China aimed to measure the emotional state of the community and associated features during the COVID-19 outbreak. Self-rating anxiety and depression scales identified high levels of emotional distress such as depression and anxiety. Further analysis on demographic variables revealed high levels of anxiety and depression among women when compared to men, and participants with higher educational qualifications showed a minimum risk of mental health problems compared to the less educated participants (Authors and Wang, 2020). Consistent with the present study, a recent study identified no gender effect on fear of COVID-19 (Levkovich et al., 2021). Consequences of another study proved the mediating role of education between fear of COVID-19 and mental health problems. Furthermore, the effect on the mental health of women was higher compared to men (Wang et al., 2020b). One more study in Italy was conducted to measure psychological distress due to COVID-19 among medical and emergency workers. This study used Emergency Stress Questionnaire (ESQ) and Traumatic Stress Scale (TSS) to measure the level of stress. The consequences of this research underlined that nurses and surgeons showed greater levels of emergency stress than medical workers working in emergency situations. Furthermore, demographics such as experience of working with COVID-19 patients, gender, and unpredicted occasions appeared as threat features for emergency stress (Vagni et al., 2020).

Existing study recruited the sample of frontline and non-frontline nurses to comparatively explore the fear of COVID-19 and emotional distress. A statistically significant difference was found in the level of fear of COVID-19 and emotional distress between frontline and non-frontline nurses. Findings suggested that the level of fear of COVID-19 and emotional distress was higher in frontline nurses compared to non-frontline nurses. A similar cross-sectional study identified a significant relationship between fear of COVID-19 and psychological distress in frontline nurses as compared to non-frontline nurses and the general population. Thus, bigger consideration should be rewarded to the emotional glitches of the health care workforce (Li et al., 2020). Moreover, a review paper identified that the extensive outbreak of COVID-19 has been linked with emotional distress and psychological ailments among people, particularly in the medical workforces and among nurses who work as frontline fighters handling the cases of COVID-19 (Jillian Mock, 2020). As nurses who worked with COVID-19 patients are directly involved in treatment, the danger of them contracting COVID-19 is greater than other medical workers. This may affect their mental state by increasing anxiety or the horror of being infected or the thought of unintentionally contaminating others. Additionally, social isolation and public separation may strengthen suspicions in nurses, causing distress to their overall mental health and job performance (Maben and Bridges, 2020). Remarkably, the prolonged use of mask contributed to social stigmatization and emotional distress related to COVID-19, since patients with pre-existent facial dermatoses experienced flares that were detrimental to the overall quality of life [use of masks related to COVID-19 increases the severity of both acne (Masked) and rosacea (mask rosacea)] (Damiani et al., 2021). Furthermore, continuous emotional distress, COVID-phobia, and social pressure also decrease the adherence to the therapies for chronic diseases (Bragazzi et al., 2020). A study of mental health of medical workers from Singapore suggested that training of healthcare workers and implementation of different psychological interventions, such as cognitive behavior therapy, can help in managing psychological distress, and people can fight the long-lasting effects of this pandemic simply by consolidating the psychological protection (Ho et al., 2020).

## Limitation

This study is based on 500 nurses from only five hospitals in Gujrat, because during the COVID-19 lockdown, only these hospitals were allowed to provide services to all the patients including COVID-19 patients in Gujrat. So, the findings could not be generalized to the staff of other hospitals. During the horrible situation of COVID-19, the healthcare staff were experiencing very tough work schedules. Hence, it was very difficult for researchers to reach the targeted population and collect data, as the participants did not cooperate for online survey which was the first option to be explored with this population.

## Conclusion

The outcome of the present study exposed that the fear of COVID-19 was a powerful predictor of emotional distress in nurses. Furthermore, the demographic features (gender, age, education, and marital status) were significant predictors of the fear of COVID-19 and emotional distress. Additionally, the comparison between frontline and non-frontline nurses indicated that frontline nurses (who worked with COVID-19 patients) displayed a high level of fear of COVID-19 and emotional distress compared to non-frontline nurses (who did not work with COVID-19 patients).

## Data Availability Statement

The original contributions presented in the study are included in the article/supplementary material, further inquiries can be directed to the corresponding author/s.

## Ethics Statement

The studies involving human participants were reviewed and approved by Advance Study and Research Board (ASRB) University of Gujrat, Gujrat, Pakistan. The patients/participants provided their written informed consent to participate in this study.

## Author Contributions

MA: conception, data collection, analysis, and write-up. MR: data collection and write-up. ZB: revision and analysis. All authors contributed to the article and approved the submitted version.

## Conflict of Interest

The authors declare that the research was conducted in the absence of any commercial or financial relationships that could be construed as a potential conflict of interest.

## Publisher's Note

All claims expressed in this article are solely those of the authors and do not necessarily represent those of their affiliated organizations, or those of the publisher, the editors and the reviewers. Any product that may be evaluated in this article, or claim that may be made by its manufacturer, is not guaranteed or endorsed by the publisher.
